# Simultaneous determination of five marker constituents in Ssanghwa tang by HPLC/DAD

**DOI:** 10.4103/0973-1296.62896

**Published:** 2010-05-05

**Authors:** Jin Bae Won, Jin Yeul Ma, Young Ran Um, Choong Je Ma

**Affiliations:** 1 *Department of Biomaterials Engineering, School of Biotechnology and Bioengineering, Kangwon National University, Chuncheon 200-701, Korea*; 2 *Research Institute of Biotechnology, Kangwon National University, Chuncheon 200-701, Korea*; 3 *TKM Converging Research Division, Korea Institute of Oriental Medicine, 483 Exporo, Yuseong-gu, Daejeon 305-811, Korea*

**Keywords:** Herbal medicine, HPLC- DAD, quality control, quantification, Ssanghwa tang

## Abstract

A HPLC-DAD method was established for the simultaneous evaluation of five bioactive compounds in Ssanghwa tang (SHT) including glycyrrhizin, paeoniflorin, cinnamic acid, decursin and 6-gingerol. These compounds were separated in less than 40 min using a Dionex C_18_ column with a gradient elution system of water and methanol at a flow rate of 1 ml/min. Calibration curve of standard components presented excellent linear regression (*R*^2^ > 0.9903) within the test range. Limit of detection and limit of quantification varied from 0.07 to 0.46 μg/ml and 0.13 to 1.11 μg/ml, respectively. The relative standard deviations (RSDs) of data of the intraday and interday experiments were less than 3.67 and 5.73%, respectively. The accuracy of recovery test ranged from 95.98 to 105.88% with RSD values 0.10– 4.82%.

## INTRODUCTION

Traditional herbal medicines are usually prepared from various herbs, and they exhibit various therapeutic effects with a complex of multiplicity components.[1,2] And the quality of these herbs has been affected by many factors such as collection time, place, temperature, cultivation environment and manufacturing process.[3–5]

This suggests the necessity of the establishment of systematic quality evaluation. In fact, quality control method of single herb has been reported earlier.[6–8] However, the quality control of traditional herbal medicinal preparation or decoction, a mixture of herb combination, has not been a research interest for a long time. Only several marker compounds were analyzed by simple HPLC analysis method. This method was fruitless as it was expensive and time consuming, and also it failed to ensure efficient quality control and standardization of traditional herbal medicine. Therefore, it is critical to develop a method for the simultaneous determination of several marker compounds in traditional herbal medicine.

Ssanghwa tang (SHT) is a traditional herbal medicinal prescription used in the treatment of infirmity, refreshment congestion, sweat, convalescence recovery, and consists of *Paeonia lactiflora*, *Glycyrrhiza glabra*, *Rehmannia glutinosa*, *Astragalus membranaceus*, *Angelica gigas*, *Zingiber officinale*, *Zizyphus jujuba*, *Cinnamomum cassia*, and *Cnidium officinale*.[9] In Korea, SHT has been commercially produced as granules by several medicinal manufactures. To ensure efficacy and safety, a suitable assay method for quality control is required.

**Figure 1:**
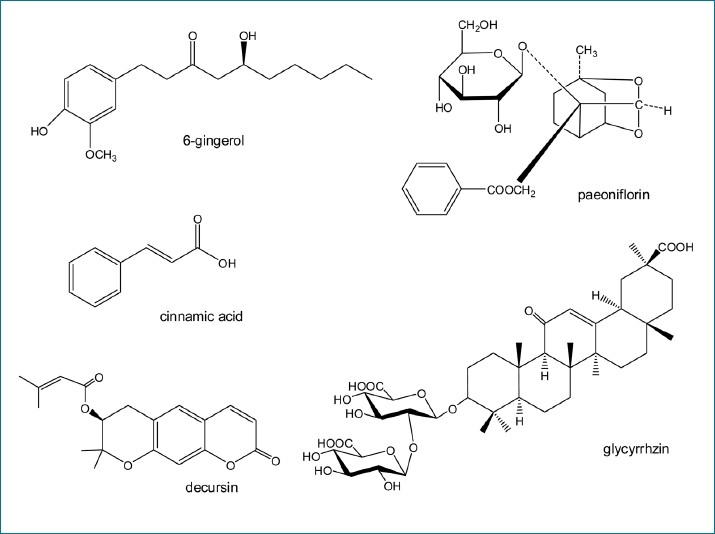
Chemical structures of five marker components in Ssanghwa tang

In this study, we employed a HPLC-DAD method for the simultaneous determination of five marker constituents in SHT, glycyrrhizin, paeoniflorin, cinnamic acid, decursin, and 6-gingerol. In addition, established analysis method was applied for the analysis of various SHT samples. The method was successfully validated and it was used to simultaneously determine five important SHT compounds [Figure 1].

## EXPERIMENTAL

### Reagents and Materials

All the five standard compounds, paeoniflorin, *trans*-cinnamic acid, glycyrrhizin, decursin and 6-gingerol, were purchased from Natural Product Chemistry BioTech Inc. (Seoul, Korea). The purity of all five marker constituents was more than 98%. All the plant materials were purchased from Kyungdong traditional herbal market (Seoul, Korea). Five commercial brands of SHT granules were purchased from local providers. HPLC grade solvents (water and methanol) and reagents were obtained from J.T. Baker (USA).

### Chromatographic conditions

Analysis was performed on the Dionex Ultimate 3000 HPLC system (Dionex, Germany) equipped with a pump (LPG 3X00), auto sampler (ACC-3000), column oven and diode array UV/VIS detector (DAD-3000(RS)). The output signal of the detector was recorded using a Dionex Chromelon™ Chromatography Data System. The separation was executed on a Dionex C_18_ column (5 μm, 120 Å, 4.6 mm× 150 mm). The mobile phase was composed of methanol (A) and water (B) with gradient elution system (0-3.5 min, 30% A isocratic; 3.5-10 min, 30-50% A; 10-30 min, 50-70% A; 30-40 min, 50% A isocratic) at a flow rate of 1.0 ml/min. The injection volume was 20 μl. The detection UV wavelength was set at 230, 254 and 280 nm. The column temperature was maintained at 25°C.

### Preparation of standard solutions and sample

Each standard stock solution was prepared by dissolving each marker components in 60% methanol at a concentration of 1 mg/ml. Each five concentrations of working solutions diluted from stock solution were used for the establishment of calibration curve. The stock solutions were stored at 4°C. For the preparation of sample, 20 mg of commercial SHT powder was accurately weighed and dissolved in 20 ml of 60% methanol. The sample solution was filtered through a 0.45 μm filter before HPLC injection.

## RESULTS AND DISCUSSION

**Figure 2:**
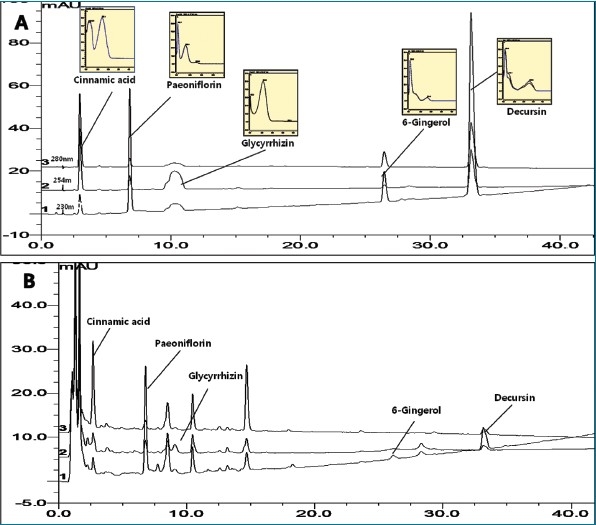
The HPLC chromatogram of standard mixture (A) and Ssanghwa tang sample (B)

The chromatographic condition was optimized to separate every peak of SHT compounds with a good resolution. We chose the Dionex C_18_ column among many reverse phase columns through the preliminary test, including a XTerra RP 18 column (250 × 4.6 mm, 5μm; Waters), LUNA C_18_ column (250 × 4.6 mm, 5μm; Phenomenex). For the simultaneous determination of the five marker constituents in SHT, glycyrrhizin, paeoniflorin, 6-gingerol, cinnamic acid and decursin, gradient solvent system of water and methanol was applied as a mobile phase. The wavelength of DAD detector was tested at 230, 254, 260, and 280 nm and set at 230 nm for peoniflorin, 6-gingerol and decursin, 254 nm for glycyrrhizin and 280 nm for cinnamic acid, where the marker compounds showed the maximum absorption as measured by a DAD detector. The presence of five marker compounds in this herbal medicine was confirmed by comparing each retention time and UV spectrum with those of each standard compound, and adding authentic standards. As a result, the optimal gradient mobile phase consisting of methanol-water was subsequently employed for the analysis of SHT, which gave good resolution and satisfactory peak shape at 230, 254, and 280 nm, respectively [Figure 2].

Specificity was confirmed by the purity of peaks detected by the diode array detector. The absorption spectrum of a single component remained little variable at each time point in one peak, which supported the specificity of each peak [Figure 2]. Our results clearly showed the specificity of each peak for five marker compounds. The linearity of five compounds was calculated based on the five concentrations of each compound. The regression equation and correlation coefficients (*R*^2^) are listed in Table 1 and high correlation coefficient values (*R*^2^ > 0.9903) showed good linearity at a relatively wide range of concentration. Limit of detection (LOD) and limit of quantification (LOQ) were determined based on the method recommended by ICH (LOD = 3.3 × (SD / slope) and LOQ = 10 × (SD / slope), SD is the standard deviation of the response, slope is the slope of calibration curve). LOD and LOQ of five marker compounds were within a range of 0.07 – 0.46 μg/ml and 0.13 - 1.11 μg/ml, respectively, which showed a high sensitivity at this chromatographic condition [Table 1].

**Table 1:** The linearity, correlation coefficient (*R*^2^), limit of detection (LOD) and limit of quantification (LOQ) of the compounds studied

Components	Linear range (μg/ml)	Regression equation^a^	*R*^2^ (*n*=5)	LOD (μg/ml)	LOQ (μg/ml)
Paeoniflorin	0.2 - 20	*Y*= 0.5074 *x* +0.1988	0.9952	0.41	0.83
6-Gingerol	0.2 - 20	*Y*=0.2015 *x* +0.0603	0.9903	0.23	0.56
Decursin	0.8 - 80	*Y*=0.4009 *x* +0.1433	0.9984	0.46	1.11
Glycyrrhizin	0.5 - 50	*Y*=0.1811 *x* +0.0897	0.9946	0.12	0.18
Cinnamic acid	0.1 - 10	*Y*=0.5770 *x* +0.0399	0.9976	0.07	0.13

a *Y*: peak area, *x*:concentration (μg/ml)

The precision test was accomplished by the intraday and interday test for these compounds. The intraday test was analyzed at three concentrations on the same day and interday test was analyzed at three concentrations on three sequential days (1, 3, 5 days). The RSD values of intraday and interday were 0.08 - 3.67% and 1.24 - 5.73%, respectively. These results indicated that this method exerted good precision [Table 2].

**Table 2:** Analytical results of intra and interday test

Components	Concentration (μg/ml)	Intraday (n=5)		Interday (n=5)	
			
		Mean±SD (μg/ml)	RSD (%)	Mean±SD (μg/ml)	RSD (%)
Paeoniflorin	4.00	4.84 ± 0.004	0.08	4.64 ± 0.11	2.31
	2.00	1.68 ± 0.04	2.08	1.51 ± 0.06	4.16
	0.20	0.13 ± 0.004	3.01	0.11 ± 0.01	2.48
6-Gingerol	4.00	4.81 ± 0.09	1.89	4.70 ± 0.07	1.49
	2.00	1.56 ± 0.02	1.25	2.10 ± 0.12	5.73
	0.20	0.20 ± 0.01	3.67	0.37 ± 0.01	3.92
Decursin	7.00	6.10 ± 0.05	0.74	5.80 ± 0.23	3.93
	3.50	3.29 ± 0.02	0.75	2.80 ± 0.10	3.41
	0.35	0.35 ± 0.01	2.23	0.25 ± 0.005	1.83
Glycyrrhizin	10.00	12.13 ± 0.25	2.03	11.64 ± 0.31	2.68
	5.00	4.05 ± 0.02	0.38	4.64 ± 0.08	1.67
	0.50	0.54 ± 0.01	1.16	0.64 ± 0.03	4.43
Cinnamic acid	2.00	2.31 ± 0.01	0.23	2.24 ± 0.06	2.52
	1.00	0.96 ± 0.01	0.63	0.92 ± 0.01	1.24
	0.10	0.15 ± 0.003	1.84	0.15 ± 0.01	4.86

The recovery test was validated by the method of spiked test. The accuracy of each compound was 95.98–105.88% with RSD values less than 4.82% (n = 3) [Table 3].

**Table 3:** Analytical results of accuracy test

Components	Spiked amount (μg/ml)	Measured amount (μg/ml)	RSD (%)	Recovery^a^ (%)
Paeoniflorin	4.00	4.00 ± 0.03	0.48	100.01
	1.50	1.51 ± 0.03	0.68	100.49
	1.40	1.37 ± 0.01	0.34	97.78
6-Gingerol	4.50	4.51 ± 1.80	1.80	100.36
	2.25	2.38 ± 0.01	1.48	105.88
	2.00	2.07 ± 0.01	2.03	103.73
Decursin	7.00	6.91 ± 0.004	0.10	98.68
	3.00	3.05 ± 0.01	2.88	95.98
	2.00	1.98 ± 0.01	0.37	99.09
Glycyrrhizin	1.50	1.58 ± 0.002	0.37	105.64
	6.50	6.30 ± 0.01	0.89	96.98
	11.00	10.62± 0.10	4.82	96.58
Cinnamic acid	2.40	2.43 ± 0.01	0.31	101.09
	1.20	1.05 ± 0.01	1.00	95.89
	1.00	1.01 ± 0.003	0.33	101.26

a Recovery (%) = (amount found − original amount)/amount spiked ×100 %

The established HPLC method was applied for the simultaneous determination of the five marker components in commercial SHT sample. Analysis result suggested that this method effectively separated marker components in SHT sample without interference of peak of other components [Table 4]. Therefore, this method was very useful to evaluate the quality control and standardization of SHT.

**Table 4:** Contents of five marker compounds in commercial Ssanghwa tang samples

	Content (mg/g)				
	
	Paeoniflorin	6-Gingerol	Decursin	Glycyrrhizin	Cinnamic acid
SHT^a^ (A)	7.95 ± 0.03	0.14 ± 0.01	3.21 ± 0.14	2.54 ± 0.43	3.16 ± 0.32
SHT (B)	3.63 ± 0.11	nd^b^	0.66 ± 0.09	2.23 ± 0.22	nd
SHT (C)	5.30 ± 0.21	nd	1.65 ± 0.32	2.67 ± 0.18	2.19 ± 0.35
SHT (D)	3.82 ± 0.14	nd	1.44 ± 0.10	2.88 ± 0.12	3.05 ± 0.21
SHT (E)	5.52 ± 0.21	nd	0.98 ± 0.14	2.53 ± 0.21	2.89 ± 0.38

a SHT: commercial Ssanghwa tang samples made by five different pharmaceutical companies,

b nd: not detected

In this study, a HPLC method was developed for the simultaneous determination of five marker components, paeoniflorin, 6-gingerol, decursin, glycyrrhzin and cinnamic acid in SHT. Validation of the method was carried out with linearity, accuracy and precision test. The results of validation indicated good precision and accuracy. The results of analysis of the commercial product suggest that this analysis method can be successfully applied for the quantification of marker compounds in SHT.
